# Supplementary material for: HYENA detects oncogenes activated by distal enhancers in cancer
Source: Nucleic Acids Res. 2024 Jul 25;52(16):e77. doi: 10.1093/nar/gkae646 (PMC11381332; doi:10.1093/nar/gkae646)
Supplement: gkae646_Supplemental_Files [file gkae646_supplemental_files.zip › HYENA.suppl.fig.docx]

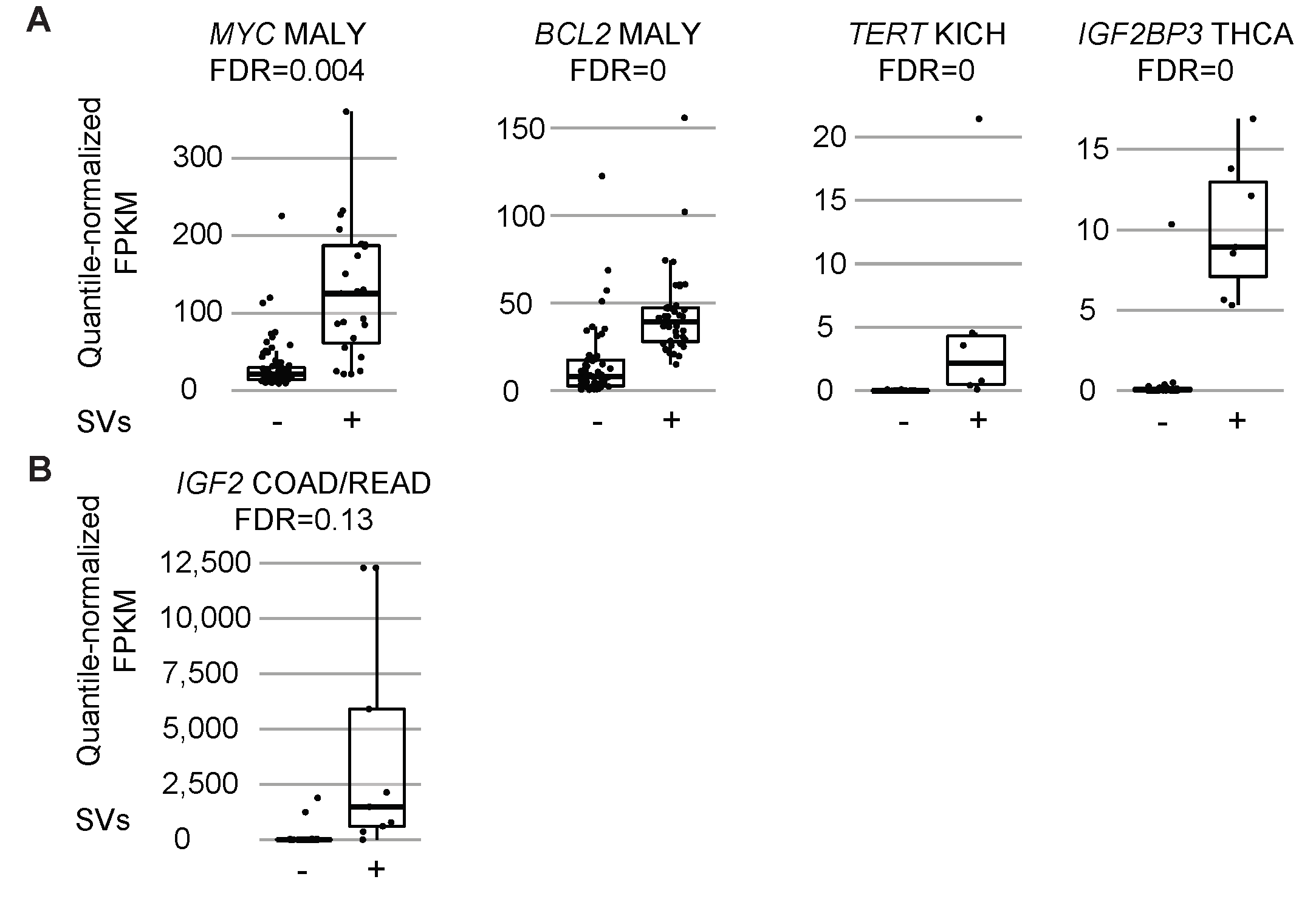


**Supplementary Fig. S1. Expression levels for five known enhancer hijacking target oncogenes.** For each gene, tumors are grouped based on SV status (- or +). Quantile normalized FPKM values are shown for each group. The boxplots show median values (thick black lines), upper and lower quartiles (boxes), and 1.5× interquartile range (whiskers). Individual tumors are shown as black dots. **A**, Genes detected by HYENA. **B**, Gene not detected by HYENA.


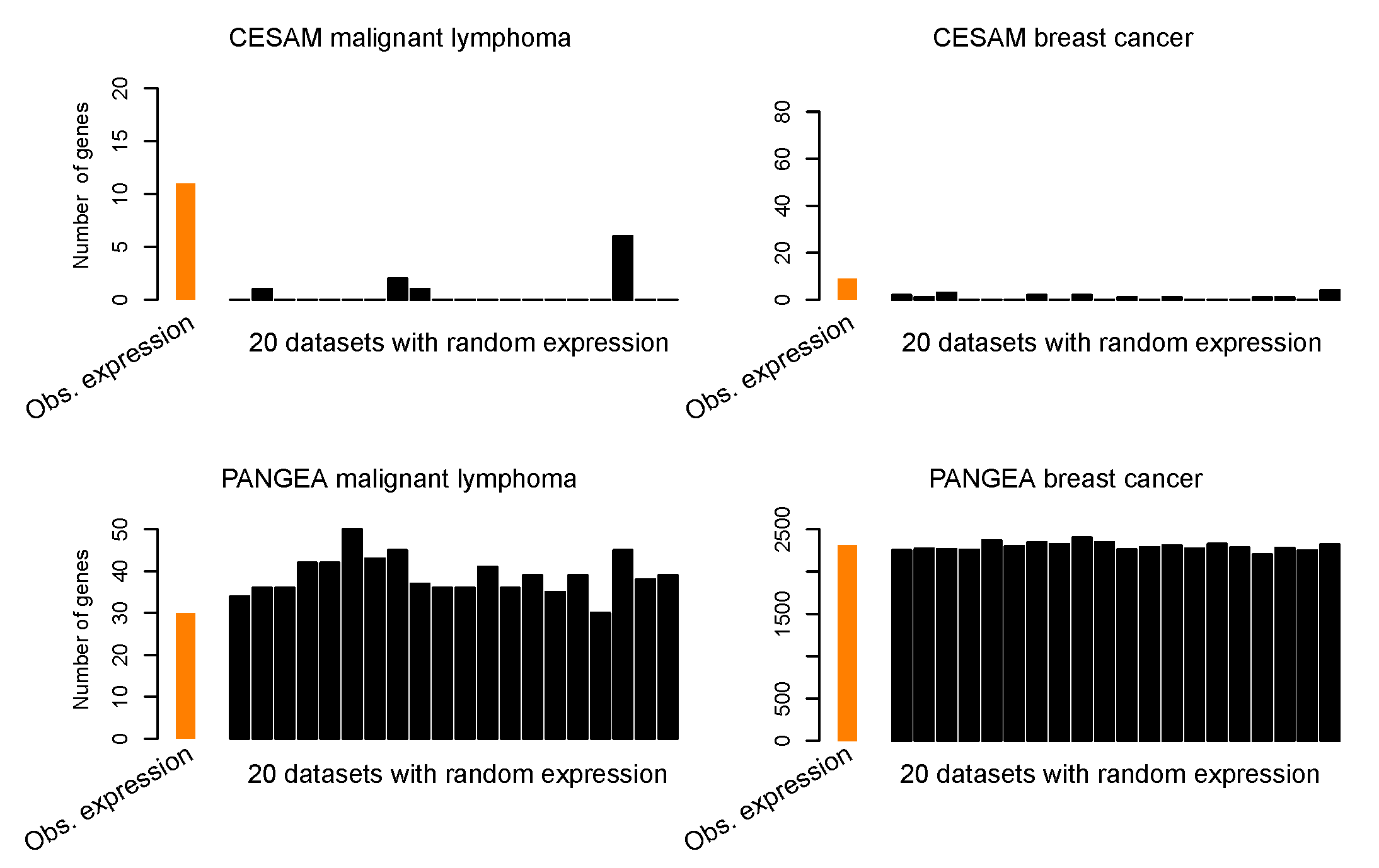


**Figure S2. Numbers of genes detected by CESAM and PANGEA in two PCAWG tumor types using observed gene expression and randomized expression.** Genes detected when expression was randomized were false positives.

**
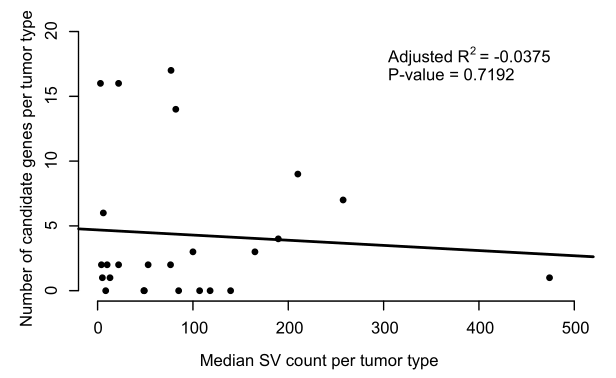
**

**Figure S3. Number of candidate enhancer-hijacking genes detected by HYENA is not associated with genome instability.** Scatter plot of median SV count and number of candidate gene detected by HYENA in each tumor type. One dot represents one tumor type. The line represents the linear regression with its statistics labeled at the upper-right corner.


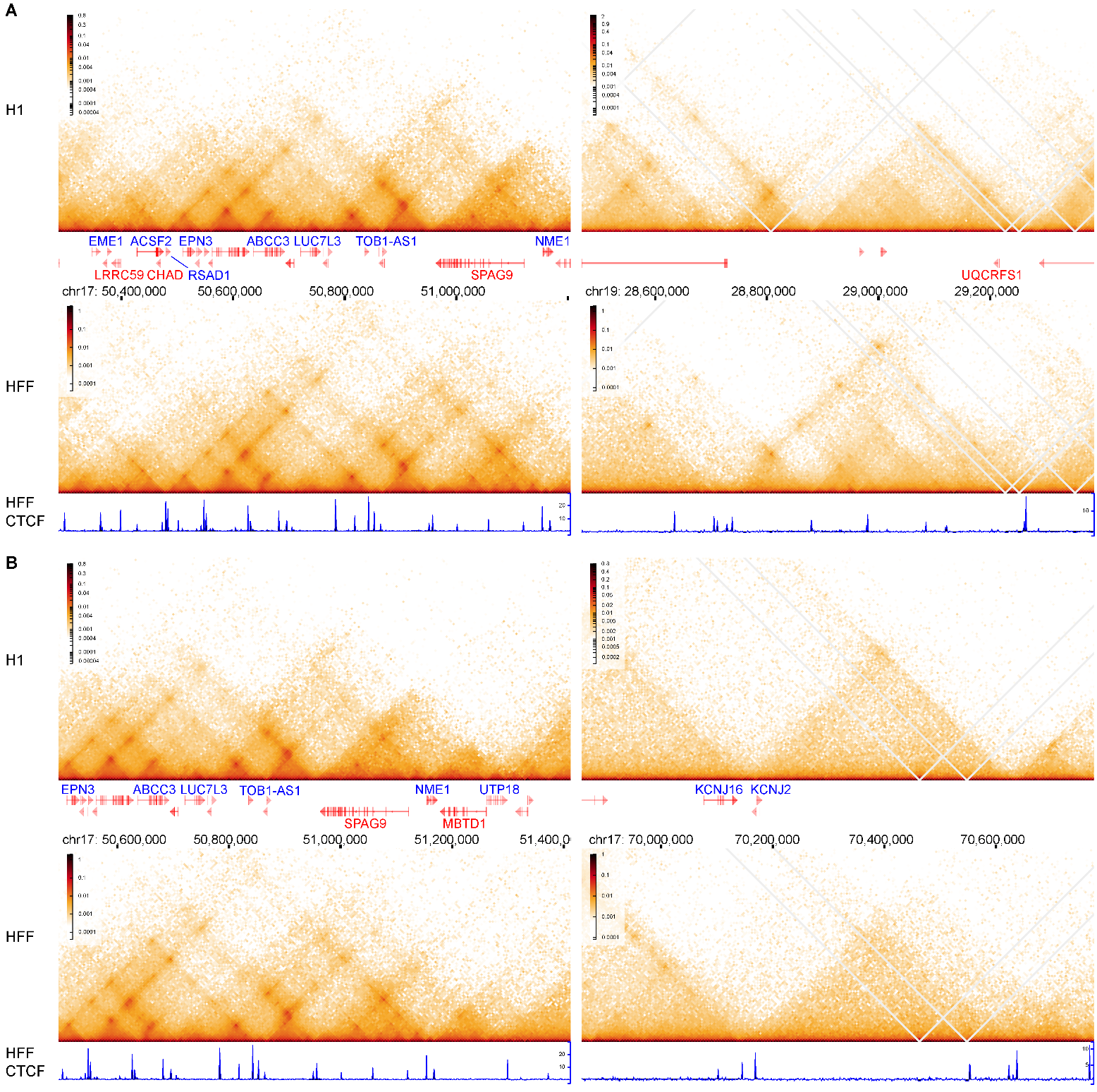


**Figure S4.** **Hi-C maps of *TOB1-AS1*, *UQCRFS1*, and *KCNJ2* loci from H1 and HFF cell lines.** **A**, *TOB1-AS1* (left panels) and *UQCRFS1* (right panels) loci. **B**, *TOB1-AS1* (left panels) and *KCNJ2* (right panels) loci. CTCF ChIP-seq of the HFF cell line is shown at the bottom. These experiment-based Hi-C maps are very similar to predicted Hi-C maps for the same loci in **Fig. 4D** and **4E** left and middle panels.


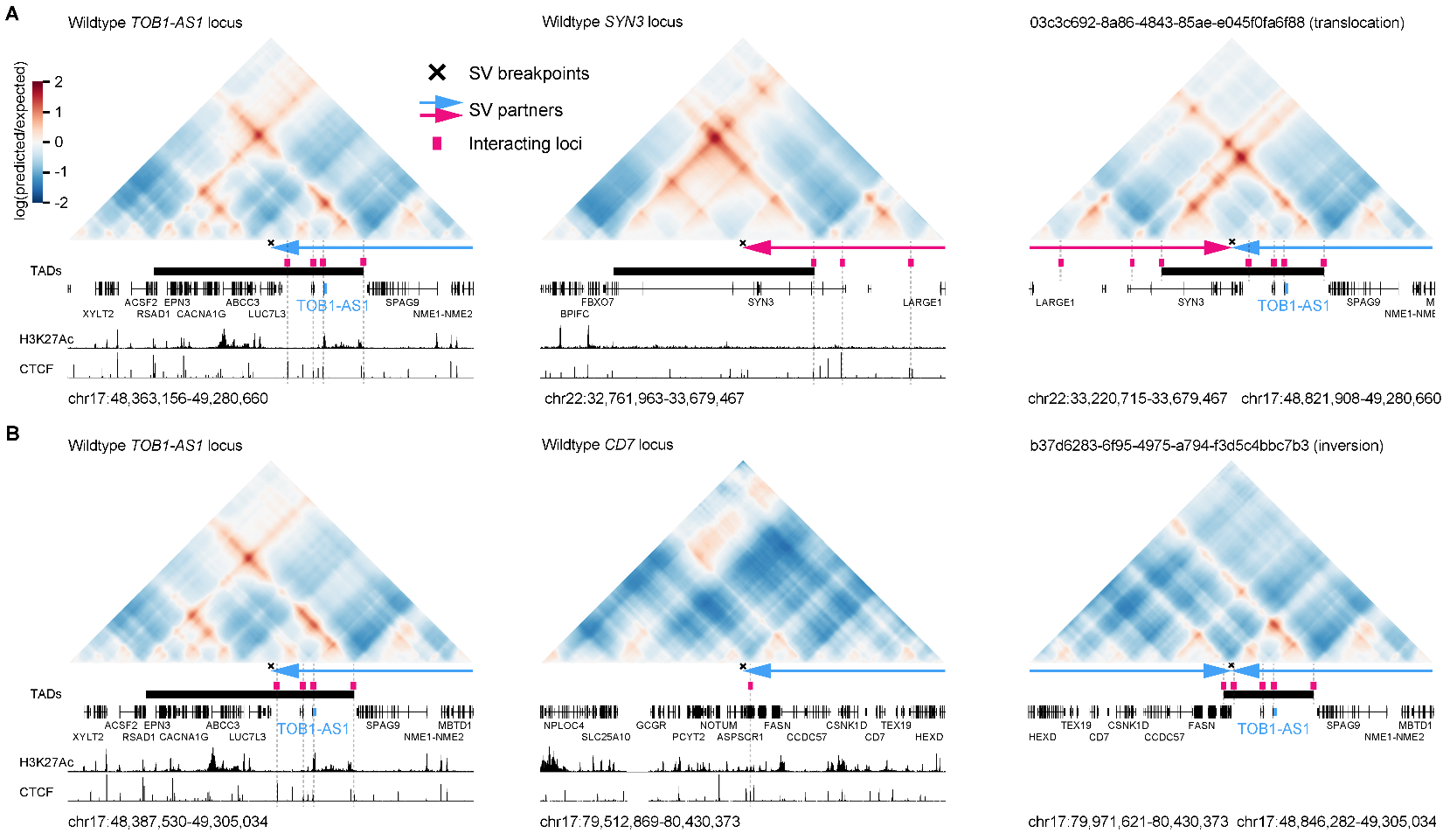


**Figure S5. Predicted 3D chromatin interaction maps for two pancreatic cancers with SVs near *TOB1-AS1*. A**, Predicted maps for regions without translocations (left and middle panels) and with translocation in tumor 03c3c692-8a86-4843-85ae-e045f0fa6f88 (right panel). **B**, Predicted maps for regions without inversion (left and middle panels) and with inversion in tumor b37d6283-6f95-4975-a794-f3d5c4bbc7b3 (right panel).


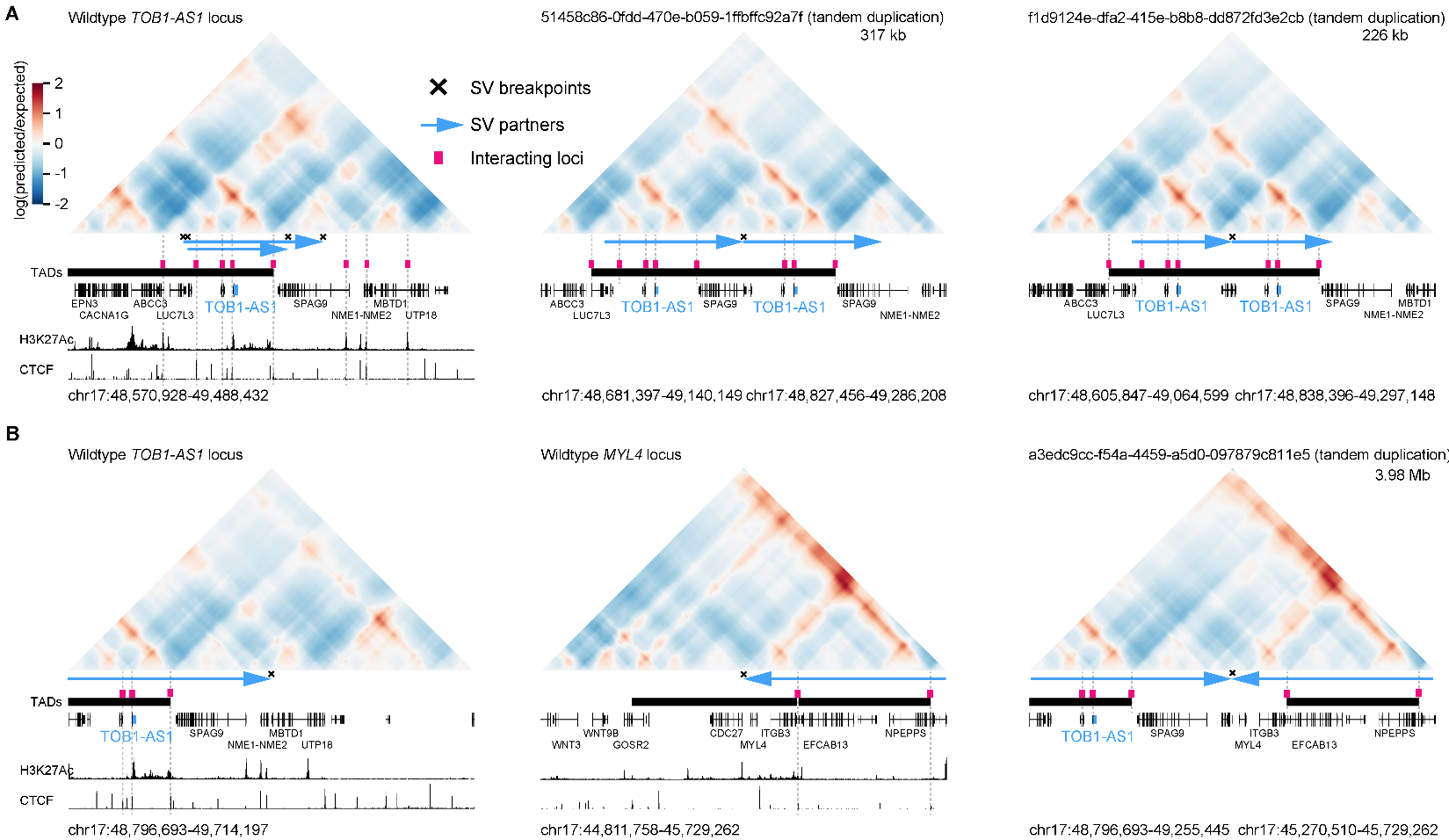


**Figure S6. Predicted 3D chromatin interaction maps for three pancreatic cancers with SVs near *TOB1-AS1*. A**, Predicted maps for regions without tandem duplication (left panel) and with tandem duplications in two tumors 51458c86-0fdd-470e-b059-1ffbffc92a7f (middle panel) and f1d9124e-dfa2-415e-b8b8-dd872fd3e2cb (right panel). **B**, Predicted maps for regions without tandem duplication (left and middle panels) and with tandem duplication in tumor a3edc9cc-f54a-4459-a5d0-097879c811e5 (right panel).


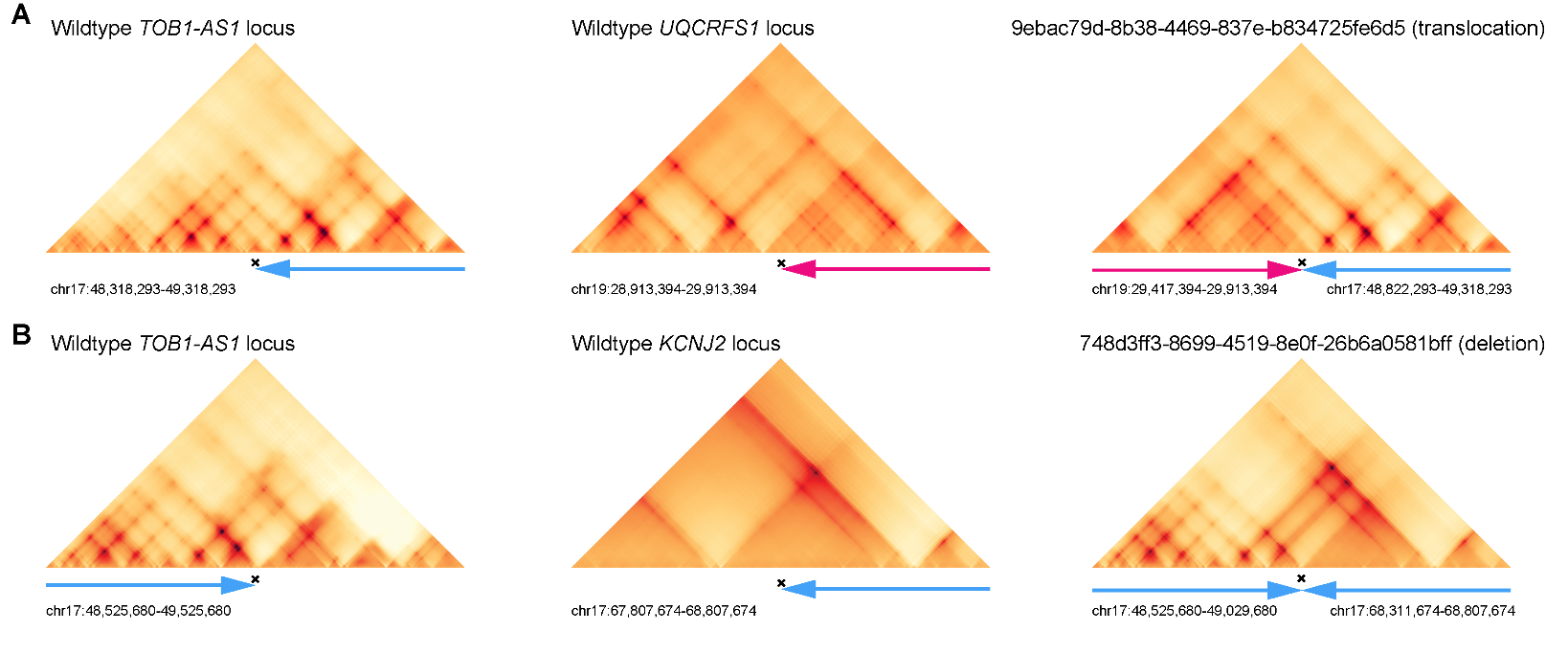


**Figure S7. 3D genome structures predicted by deep-learning based algorithm Orca.** **A**, Predicted 3D chromatin interaction maps of the *TOB1-AS1* (left panel), *UQCRFS1* (middle panel), and the translocated region in tumor 9ebac79d-8b38-4469-837e-b834725fe6d5 (right panel). **B**, Predicted 3D chromatin interaction maps of *TOB1-AS1* (left panel) and *KCNJ2* (middle panel) loci without deletion as well as the region after deletion in tumor 748d3ff3-8699-4519-8e0f-26b6a0581bff (right panel). The 6 regions in this figure are the same regions shown in **Fig. 4D** and **4E**.


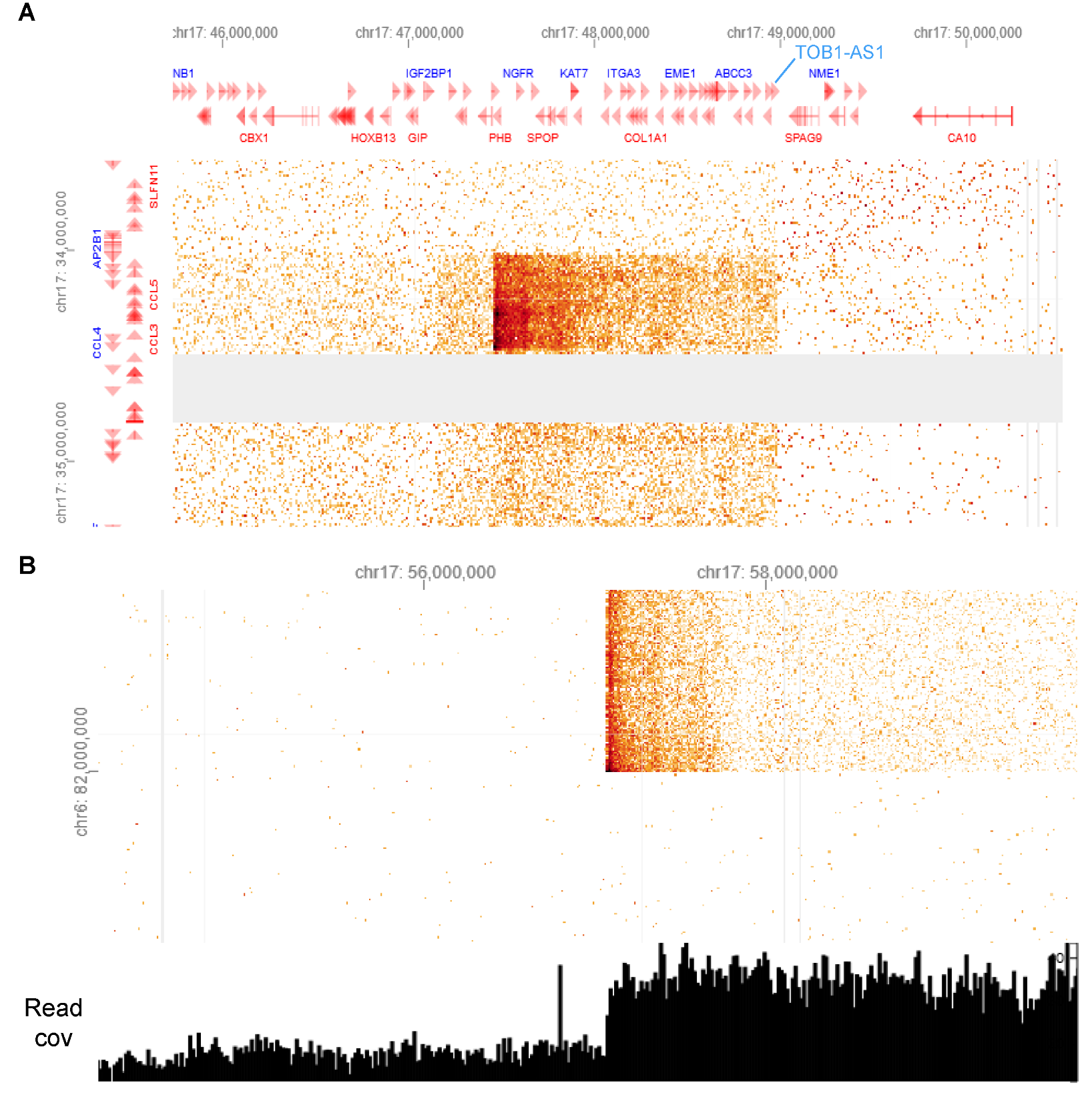


**Figure S8. SVs in Panc 10.05 detected by Hi-C.** **A**, HiGlass view showing a deletion of chr17:34,460,000-47,450,000. **B**, HiGlass view showing a translocation between chromosomes 6 and 17. Read coverage is shown below the Hi-C contact map. The chromosome 17 translocation breakpoint is 8 Mb downstream of the CNV breakpoint shown in **Fig. 5C** left most panel.


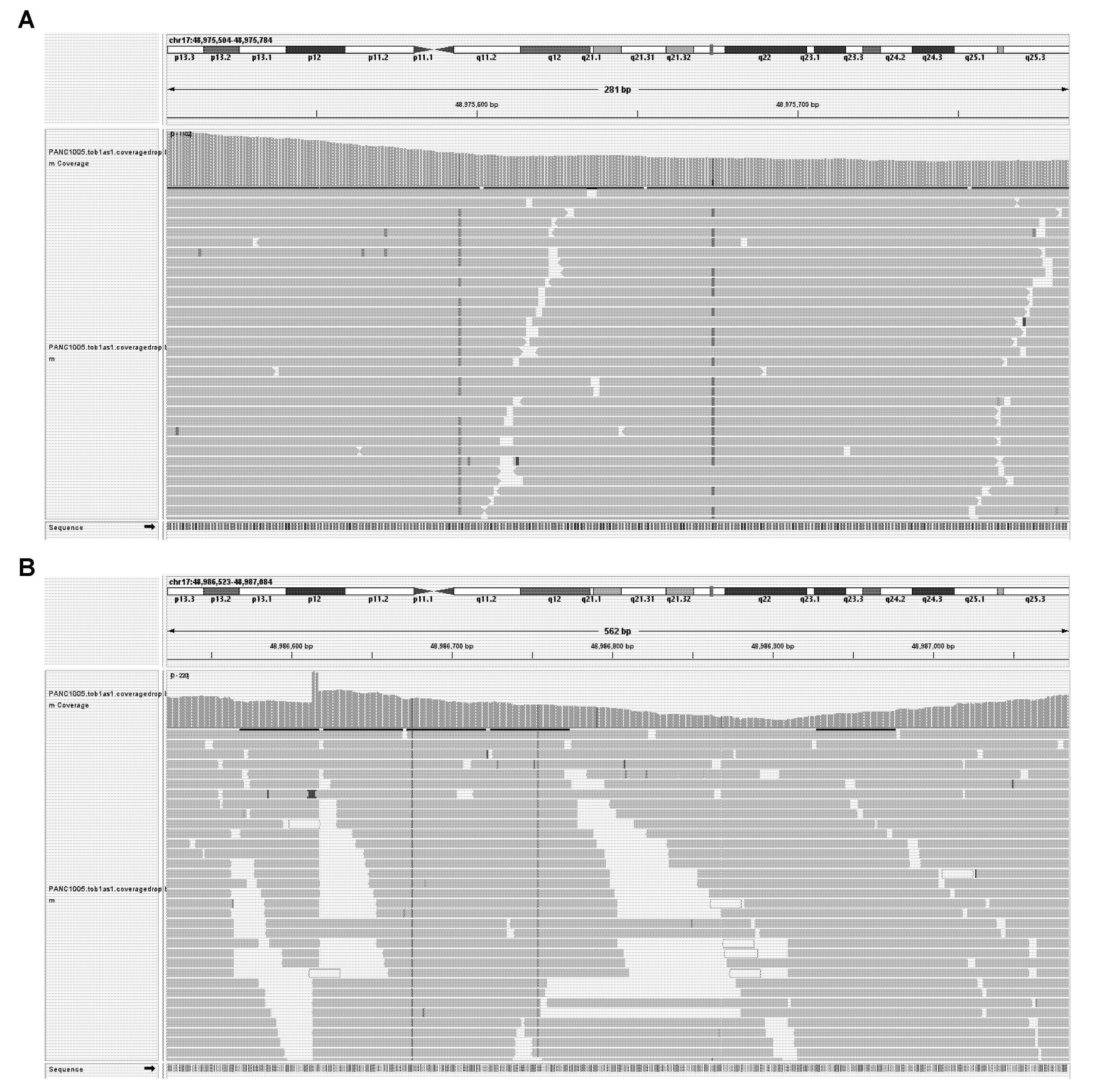


**Figure S9. SNPs in Panc 10.05 near CNV and foldback inversion breakpoint.** **A** and **B**, IGV screenshots showing reads mapped to five-copy and one-copy regions in Panc 10.05 in **Fig. 5C** left most panel. Horizontal grey bars are Hi-C sequencing reads. Colored lines are mismatches of reads compared to the reference genome. Grey vertical bars are read depth. Colored vertical bars represent SNPs. The two SNPs in **A** are heterozygous SNPs, whereas the four in **B** are homozygous.


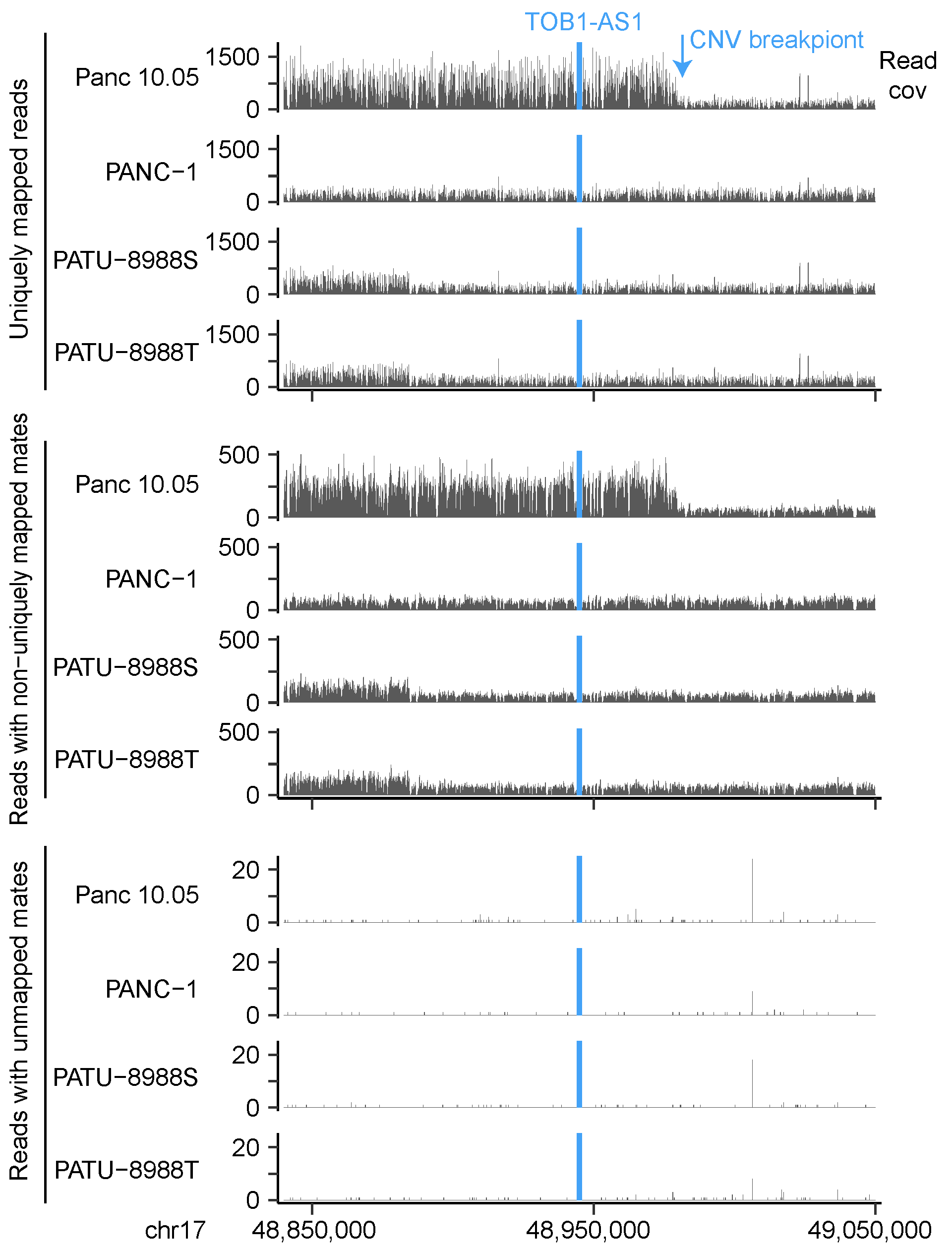


**Figure S10. Read coverage in 4 pancreatic cell lines at the *TOB1-AS1* locus.** The numbers of uniquely mapped reads, reads with non-uniquely mapped mates, and reads with unmapped mates are shown for four pancreatic cancer cell lines. In Panc 10.05, the ratio of non-uniquely mapped mates in the high-copy region (upstream of the CNV breakpoint) and the low-copy region (downstream of the CNV breakpoint) is comparable to that of uniquely mapped reads. Very few unmapped mates are present in any cell lines.


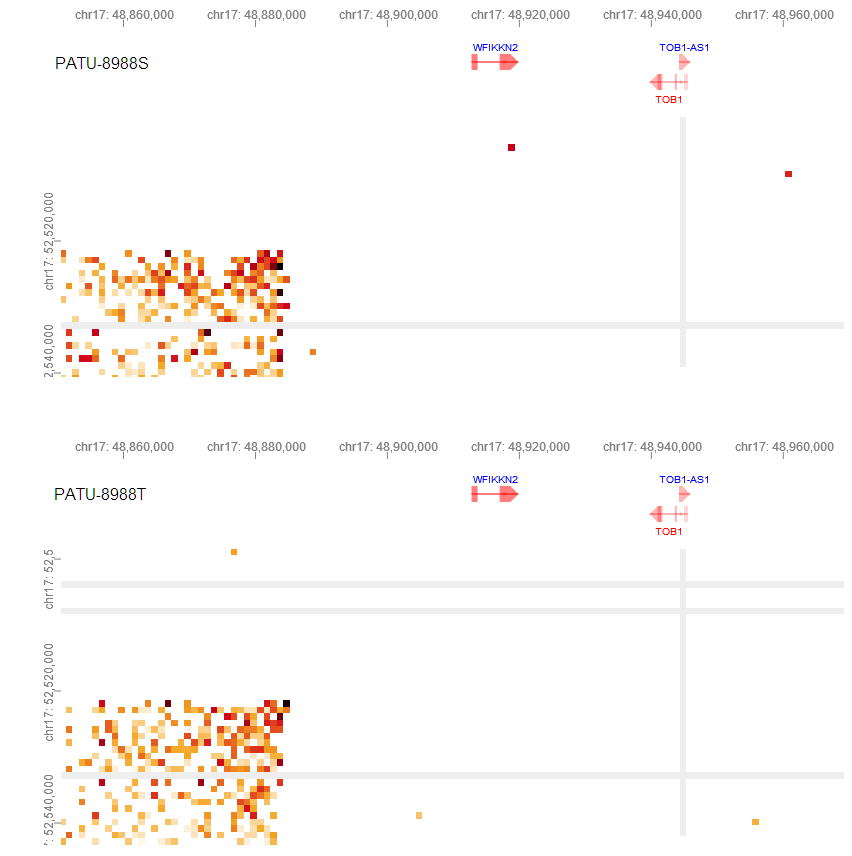


**Figure S11. HiGlass views showing the shared SV near *TOB1-AS1* in PATU-8988S (top) and PATU-8988T (bottom).** The SV is about 50 kb upstream of *TOB1-AS1* and points away from *TOB1-AS1*. The locations of *TOB1-AS1* are shown in the x-axis at the top.

**
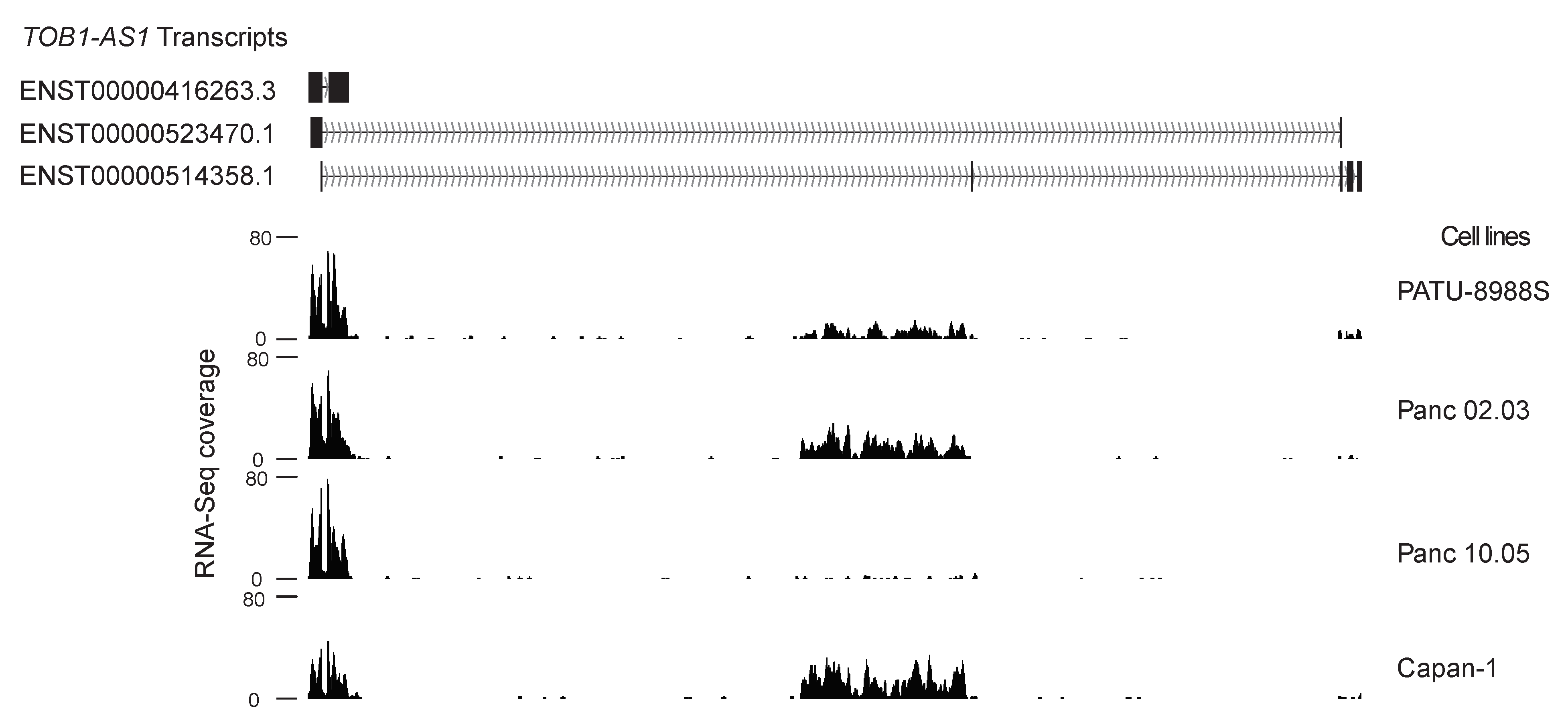
**

**Figure S12. RNA-Seq coverage of *TOB1-AS1* isoforms.** RNA-Seq coverage of three *TOB1-AS1* isoforms from four pancreatic cancer cell lines with high *TOB1-AS1* expression (PATU-8988S, Panc 02.03, Panc 10.05, and Capan-1). The major isoform is ENST00000416263.3.

**Figure S13. *TOB1-AS1* overexpression.** **A**, *TOB1-AS1* relative expression levels in PATU-8988T, PANC-1, Panc 10.05, and PATU-8988S cell lines based on quantitative RT-PCR. The relative expression of the other three cell lines was calculated relative to PATU-8988T. Error bars indicate standard error of the mean. **B**, Ex vivo IVIS images showing primary tumors and spleen metastatic tumors from mice orthotopically injected with PANC-1. **C**, Ex vivo IVIS images and radiance quantification (p/sec) of whole wells showing liver metastatic tumors in mice orthotopically injected with PANC-1. Two-sided student t test was used. Error bars indicate the standard error of the mean. **D**, Scatter plots showing the correlations between *TOB1* and *TOB1-AS1* RNA expression in CCLE pancreatic cancer cell lines, TCGA PAAD, PCAWG PACA-AU, and ICGC PACA-CA cohorts. Sample sizes, gene expression normalization methods, squared-Rs and *P* values are labeled. In CCLE cell lines and the PCAWG PACA-AU cohort, the two genes have very weak positive associations with marginal *P* values of 0.029 and 0.027. In the ICGC PACA-CA cohort, the two genes have a strong positive correlation. However, the correlation is mainly driven by two outliers. On the contrary, in the TCGA PAAD cohort, the two genes are not significantly correlated. Therefore, *TOB1-AS1* and *TOB1* do not have consistent associations in patient samples and cell lines. **E**, Volcano plot showing the differentially expressed genes in *TOB1-AS1* overexpression PANC-1 tumors (n=6) compared to vector control tumors (n=6). Red and blue dots with gene labels represent significantly (FDR <0.1) upregulated and downregulated genes with fold-change larger than 1.5 and smaller than 1/1.5, respectively. Grey dots represent all other genes. Grey lines represent -log10(FDR) of 1 (horizontal), log2(FoldChange) of log2(1.5) (vertical, right) and log2(1/1.5) (vertical, left).
